# Reliability of gastrointestinal barrier integrity and microbial translocation biomarkers at rest and following exertional heat stress

**DOI:** 10.14814/phy2.14374

**Published:** 2020-03-13

**Authors:** Henry B. Ogden, Joanne L. Fallowfield, Robert B. Child, Glen Davison, Simon C. Fleming, Robert M. Edinburgh, Simon K. Delves, Alison Millyard, Caroline S. Westwood, Joseph D. Layden

**Affiliations:** ^1^ School of Sport, Health and Wellbeing Plymouth MARJON University Plymouth United Kingdom; ^2^ Institute of Naval Medicine Alverstoke United Kingdom; ^3^ School of Chemical Engineering University of Birmingham Birmingham United Kingdom; ^4^ Endurance Research Group School of Sport and Exercise Sciences University of Kent Chatham Maritime United Kingdom; ^5^ Royal Cornwall NHS Trust Truro United Kingdom; ^6^ Department of Health University of Bath Bath United Kingdom

**Keywords:** endotoxin, exercise, gut

## Abstract

**Purpose:**

Exertional heat stress adversely distrupts (GI) barrier integrity and, through subsequent microbial translocation (MT), negativly impacts health. Despite widespread application, the temporal reliability of popular GI barrier integity and MT biomarkers is poorly characterised.

**Method:**

Fourteen males completed two 80‐min exertional heat stress tests (EHST) separated by 7–14 days. Venous blood was drawn pre, immediately‐ and 1‐hr post both EHSTs. GI barrier integrity was assessed using the serum Dual‐Sugar Absorption Test (DSAT), Intestinal Fatty‐Acid‐Binding Protein (I‐FABP) and Claudin‐3 (CLDN‐3). MT was assessed using plasma Lipopolysaccharide Binding Protein (LBP), total 16S bacterial DNA and *Bacteroides* DNA.

**Results:**

No GI barrier integrity or MT biomarker, except absolute *Bacteroides* DNA, displayed systematic trial order bias (*p* ≥ .05). I‐FABP (trial 1 = Δ 0.834 ± 0.445 ng ml^−1^; trial 2 = Δ 0.776 ± 0.489 ng ml^−1^) and CLDN‐3 (trial 1 = Δ 0.317 ± 0.586 ng ml^−1^; trial 2 = Δ 0.371 ± 0.508 ng ml^−1^) were increased post‐EHST (*p* ≤ .01). All MT biomarkers were unchanged post‐EHST. Coefficient of variation and typical error of measurement post‐EHST were: 11.5% and 0.004 (ratio) for the DSAT 90‐min postprobe ingestion; 12.2% and 0.004 (ratio) at 150‐min postprobe ingestion; 12.1% and 0.376 ng ml^−1^ for I‐FABP; 4.9% and 0.342 ng ml^−1^ for CLDN‐3; 9.2% and 0.420 µg ml^−1^ for LBP; 9.5% and 0.15 pg µl^−1^ for total 16S DNA; and 54.7% and 0.032 for *Bacteroides*/total 16S DNA ratio.

**Conclusion:**

Each GI barrier integrity and MT translocation biomarker, except *Bacteroides*/total 16S ratio, had acceptable reliability at rest and postexertional heat stress.

## Graphical abstract

The aim of this study was to assess the reliability of gastrointestinal barrier integrity and microbial translocation biomarkers both at rest and in response to exertional heat stress. Fourteen healthy male participants undertook two identical exertional heat stress tests interspersed by 7–14 days. Acceptable and defined levels of reliability are presented for the serum Dual‐Sugar Absorption (lactulose/L‐rhamnose) Test, Intestinal Fatty‐Acid Binding Protein, Claudin‐3, Lipopolysaccharide Binding Protein, total 16s DNA, but not the *Bacteroides*/total 16s DNA ratio at both measurement points.

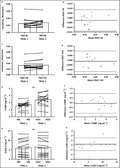

## 1 INTRODUCTION

The gastrointestinal (GI) microbiota is a complex microbial ecosystem, which performs numerous functions symbiotic to human health (Cani, 2018). However, to prevent immune activation, the microbiota must remain contained within the GI lumen, a process that is tightly regulated by the multilayered GI barrier (Wells et al., 2017). Exertional heat stress is one stimulus that adversely disrupts GI barrier integrity, and in a linear manner to the severity of splanchnic hypoperfusion (van Wijck et al., 2011) and core body temperature (Pires et al., 2017). In severe cases, luminal microbial products are capable of transversion into the systemic circulation, a response now considered to underlie multiple common athletic health conditions (Costa, Snipe, Kitic, & Gibson, 2017). Specifically, the most concerning of these health conditions include exercise‐induced anaphylaxis (Christensen et al., 2019) and exertional heatstroke (Lim, 2018). In nonexercise settings, research has linked GI microbial translocation (MT) within the pathophysiology of numerous chronic diseases, including GI disease (Camilleri, Madsen, Spiller, Meerveld, & Verne, 2012), cardiovascular disease (Neves, Coelho, Couto, Leite‐Moreira, & Roncon‐Albuquerque, 2013), and degenerative disorders of the central nervous system (Mulak & Bonaz, 2015). Thus, reliable biomarkers of GI barrier integrity and/or MT appear important in the surveillance, diagnosis and treatment of these conditions. To date, there is little evidence documenting the reliability of most commonplace biomarkers, which limits interpretation of their application in both laboratory and field settings.

GI barrier integrity can be assessed in vivo using several biomarkers of intestinal permeability, epithelial injury, and tight junction integrity (Wells et al., 2017). The Dual‐Sugar Absorption Test (DSAT) is the gold standard GI permeability biomarker (Bischoff et al., 2014). The traditional endpoint of the DSAT is the 5‐hr urinary recovery of pre‐ingested lactulose‐to‐_L_‐rhamnose (L/R; Bischoff et al., 2014) and offers good test–retest reliability when applied at rest (Marchbank et al., 2010). Analytical improvements have recently permitted validation of a serum DSAT over a reduced (i.e., 1–3 hr) time course (van Wijck et al., 2013) and with improved diagnostic sensitivity (JanssenDuijghuijsen et al., 2016; Pugh et al., 2017). However, given the time course appearance of sugar probes within the blood (Fleming, Duncan, Russell, & Laker, 1996), potentially due to the wide heterogeneity in gastric emptying rates following exercise (Costa et al., 2017), the reliability of this biomarker requires verification. Intestinal Fatty Acid‐Binding Protein (I‐FABP) is a cytosolic protein expressed exclusively within enterocytes of the duodenum/jejunum, and has a half‐life of 11 min in the systemic circulation following epithelial injury (van de Poll et al., 2007). These characteristics have popularized I‐FABP as a prominent biomarker of small GI epithelial injury (Wells et al., 2017), with serum concentrations strongly predictive of small GI histological injury (Schellekens et al., 2014). The temporal reliability of I‐FABP has never been directly assessed and requires interrogation given its high sensitivity to subclinical small GI injury. Claudin‐3 (CLDN‐3) is a conserved GI epithelial transmembrane protein, which performs an integral role in GI paracellular homeostasis (Zeissig et al., 2007). As a biomarker of GI tight junction (TJ) integrity, preliminary research has shown a strong relationship between urinary CLDN‐3 concentration and histological GI CLDN‐3 breakdown (Thuijls, Derikx, Haan, et al., 2010a; Thuijls, Derikx, van Wijck, et al., 2010b). Similar to I‐FABP, the temporal reliability of plasma CLDN‐3 is currently unknown.

GI MT can be assessed in vivo through several indirect biomarkers considered to be indicative of systemic microbial exposure (Wells et al., 2017). Endotoxin, a form of lipopolysaccharide located on the outer membrane of gram‐negative bacteria, has traditionally been utilized for this purpose (Costa et al., 2017). However, the search for improved GI MT biomarkers is ongoing, given endotoxin analysis is susceptible to both false‐positive (e.g., from exogenous contamination) and false‐negative (e.g., from rapid hepatic clearance) results (Dullah & Ongkudon, 2017). Lipopolysaccharide‐binding protein (LBP) is a type‐1 acute phase protein, secreted hepatically following systemic exposure to numerous microbial‐associated molecular patterns (Schumann, 2011). However, as an acute‐phase protein, its temporal reliability is likely highly subject to influence from numerous co‐variates (e.g. infection) (Citronberg et al., 2016). Bacterial DNA (bactDNA), through conserved 16S gene sequencing, is an emerging biomarker of GI MT (Paisse et al., 2016). In comparison with alternative MT measures, one major advantage of bactDNA is an apparent independence of hepatic clearance (Mortensen et al., 2013). One innovative study recently proposed a bactDNA methodology aimed to improve analytical specificity and reliability through targeting a predominant GI bacterial genus (*Bacteroides*) and correcting for total 16S DNA concentration (March, Jones, Thatcher, & Davison, 2019).

The aim of this study was to determine the reliability of biomarkers of GI barrier integrity (DSAT, I‐FABP, CLDN‐3) and microbial translocation (LBP, total 16s bacterial DNA, *Bacteroides* DNA) at rest and following exertional heat stress. These data should inform prospective research in this field, including biomarker selection and statistical power.

## 2 METHODS

### 2.1 Participants and ethical approval

Fourteen healthy males (Table 1) volunteered to participate in the present study. All participants were nonsmokers, habitually active, nonendurance trained (>4 hr week^−1^) and unacclimated to hot environments. A general medical questionnaire was used to screen for previous histories of gastrointestinal, cardiorespiratory, and metabolic illnesses. No participant took pharmacological medications (e.g., laxatives, antibiotics) or reported suffering from an acute illness within 14 days prior to data collection. Informed consent was obtained for each participant following a full written and oral explanation of the experimental procedures. The study protocol was approved by Plymouth MARJON University Research Ethics Committee (Approval Code: EP040) and was conducted in accordance with the principles outlined in the Declaration of Helsinki, except for trial registration within a database.

**Table 1:** Participant demographic characteristics

Measure	Mean ± *SD*
Age (years)	26 ± 5
Height (m)	1.78 ± 0.06
Body Mass (kg)	83.4 ± 12.6
Physical Activity (h·week^−1^)	6 ± 3
Body Fat (%)	16.1 ± 4.0
V̇O_2max_ (ml·kg^−1^·min^−1^)	49 ± 4

### 2.2 Experimental overview

**Figure 1:**
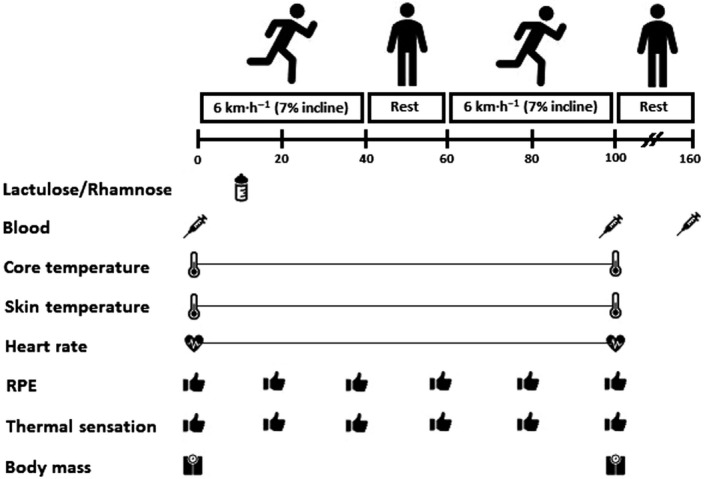
Schematic illustration of the experimental design and measurement timings

Participants visited the laboratory on three occasions. During the first visit, baseline anthropometrics and maximal oxygen uptake (V̇O_2max_) were assessed. The second and third visits were the main experimental trials. These were separated by 7–14 days to negate the influence of prior exertional heat stress on thermoregulatory (Barnett & Maughan, 1993) and GI barrier integrity (Snipe, Khoo, Kitic, Gibson, & Costa, 2017) responses. During both main experimental trials, participants completed an intermittent exertional heat stress test (EHST), consisting of two bouts of 40 min fixed‐intensity treadmill walking (6 km h^−1^ and 7% gradient) in the heat (35°C and 30% relative humidity; RH). The exercise bouts were separated by 20‐min seated recovery in the heat, including 4‐min forearm cold water immersion. This protocol is consistent with general military guidance on work/rest schedules for sustained physical activity in the heat (Military Headquarters of the Surgeon General, 2017) and unpublished pilot data from our laboratory showing a ~2‐fold elevation in DSAT responses relative to rest (*n* = 6; DSAT 90‐min postprobe ingestion; [rest] = 0.014 ± 0.006, [post‐EHST] = 0.028 ± 0.005; *p* = .02). Data collection coincided with nonsummer months in Plymouth, United Kingdom, where daily mean ambient temperature at a local meteorological station (Camborne, United Kingdom; latitude: 50.218°N) remained below 20°C (Met Office, 2019). A schematic illustration of the protocol is shown in Figure 1.

### 2.3 Dietary and lifestyle controls

Dietary supplementation (e.g., glutamine, probiotics, bovine colostrum) and prolonged thermal exposures (e.g., saunas, sunbeds) were prohibited from 14 days before until the end of data collection (Costa et al., 2017). Alcohol, caffeine, strenuous physical activity and nonsteroidal anti‐inflammatory drugs (e.g., ibuprofen) were all abstained for 48 hr before main experimental visits (Costa et al., 2017). Participants adhered to a ≥10 hr overnight fast and consumed 500 ml of plain water 2 hr prior to main experimental visits. Conformity with all pretrial controls was assessed in writing upon laboratory arrival using a pretrial control questionnaire. Participants were fasted throughout all main experimental trials (Edinburgh et al., 2018), but permitted 12 ml kg^−1^ of ambient temperature water (28°C–30°C) to drink over the 20 min recovery following both 40‐min EHST bouts.

### 2.4 Anthropometric measurements

Participants height, weight, and body fat were measured following ISAK guidelines (Marfell‐Jones, Olds, Stewart, & Carter, 2006). Height was measured barefoot using a stadiometer to the nearest 0.1 cm (Marsden HM‐200), whilst body mass was measured on an electronic scale to the nearest 0.05 kg (Tanita MC 180 MA). Skinfold thicknesses were taken in duplicate by the same researcher at the bicep, tricep, subscapular, and suprailliac using skinfold callipers to the nearest 0.1 cm (Harpenden, Holtain Ltd). Predictions of body density were calculated using age and gender‐related equations (Durnin & Womersley, 1974).

### 2.5 Maximal oxygen uptake

Maximal oxygen uptake (V̇O_2max_) was determined using an incremental treadmill test (Desmo HP, Woodway GmbH, Weil am Rhein, Germany) to volitional exhaustion. The test was undertaken in normothermic laboratory conditions (18°C–22°C, 40%–60% RH). The test began at a speed of 10 km h^−1^ on a fixed 1% inclination. The treadmill speed was then increased at 1 km h^−1^ increments every three minutes until reaching 13 km h^−1^, when inclination was then increased by 2% every 2 min. Expired metabolic gases were measured continuously using a breath‐by‐breath metabolic cart (Metalyser 3B, Cortex). Heart rate (HR; Polar FT1, Polar Electro OY) and rating of perceived exertion (RPE; Borg, 1970) were measuring during the final ten seconds of each stage. The highest 30 s average V̇O_2_ was taken to be V̇O_2max_.

### 2.6 Exertional heat stress test

EHSTs commenced in the morning (08:30 ± 1 hr) to avoid the influence of circadian variation (Waterhouse et al., 2005). Upon laboratory arrival, participants provided a capillary blood sample into a K_2_EDTA microtube (Microvette®, Sarstedt) for duplicate hydration assessment via plasma osmolality using freeze‐point depression (Osmomat 3000, Gonotec). Participants then measured their own nude body mass (Tanita MC 180 MA). They then self‐inserted a single use rectal thermistor (T_core_; Phillips 21090A) 12 cm beyond the anal sphincter and a HR monitor was positioned around their chest (EQ02, Equivital™). Participants then dressed in standard summer military clothing (i.e., jacket [zipped, sleeves extended], trousers, boxer briefs, socks, trainers), and entered the environmental chamber that was regulated at ~ 35°C (Trial 1: 35.2 ± 0.3°C; Trial 2: 35.4 ± 0.4°C; *p* = .15) and ~ 30% RH (Trial 1: 28 ± 4%; Trial 2: 28 ± 2%; *p* = .25). Skin thermistors (EUS‐UU‐VL3‐O, Grant Instruments) were then affixed on the participant using one layer (5 × 5 cm) of cotton tape (KT Tape®, KT Health) and mean skin temperature (T_skin_) was calculated using standard equations (Ramanathan, 1964).

Participants then undertook the predefined EHST. Throughout, T_core_ and T_skin_ were recorded using a temperature logger (Squirrel SQ2010, Grant Instruments) and HR using a Sensor Electronics Module (*SEM*) unit (EQ02, Equivital™). Mean whole body temperature (T_body_) was calculated from simultaneous T_core_ and T_skin_ measurements (Jay & Kenny 2007). All data, including RPE (Borg et al., 1970) and thermal sensation (TS; Toner, Drolet, & Pandolf, 1986) were reported at 20‐min intervals. Between the two walking bouts, participants immersed their forearms in a ~15°C cold water bath (Trial 1: 15.4 ± 0.8°C, Trial 2: 15.3 ± 0.7°C; *p* = .39). Upon EHST termination, participants were removed from heat and their post‐EHST nude body mass was recorded. Absolute sweat losses were calculated from the change in dry nude body mass from pre‐to‐post‐EHST after correction for fluid intake and blood withdrawal.

### 2.7 Blood collection and analysis

Venous blood samples (12 ml) were drawn immediately pre‐, post‐ and 1‐hr post‐EHST. Participants stood upright for a minimum of 20 min before collection to allow capillary filtration pressure to stabilise (Shirreffs & Maughan, 1994). Blood was drawn from a forearm antecubital vein under minimal stasis (<30 s). Samples were collected proportionally into serum separator (SST II) and K_2_ EDTA tubes (Becton Dickinson and Company). The SST II tube was allowed to clot for 30–40 min at room temperature. A 0.5 ml aliquot of K_2_EDTA blood was removed for immediate hematological analysis. Samples were centrifuged at 1300 *g* for 15 min at 4°C to separate serum and plasma. Aliquots were frozen at −80°C until analyses. All blood handling was performed with sterile (pyrogen, DNA, RNA free) pipette tips and microtubes.

### 2.8 Hematology

Hemoglobin was measured in duplicate using a portable photometric analyser (Hemocue® Hb 201+, EFK Diagnostics; Duplicate) and hematocrit in duplicate using the microcapillary technique following centrifugation at 14,000 *g* for 4 min at room temperature (Haematospin 1400, Hawksley and Sons Ltd). Plasma volume was estimated using standard equations (Dill & Costill, 1974). Postexercise analyte concentrations were left uncorrected for acute plasma volume shifts, given the similarity of responses between trials and the low molecular weights of quantified analytes.

### 2.9 Dual‐Sugar Absorption Test

Participants orally ingested a standard sugar probe solution containing 5 g Lactulose (Lactulose Oral Solution, Sandoz) and 2 g _L_‐Rhamnose (_L_‐rhamnose FG, 99% pure, Sigma Aldrich) dissolved within 50 ml of plain water (osmolality = ~750 mOsm·kg^‐1^) 10 min into the EHST. Probe concentrations were determined from serum samples collected immediately pre, 90 min (i.e., post‐EHST) and 150 min (i.e., 1‐hr post‐EHST) post‐probe ingestion following a previously described high performance liquid chromatography protocol (Fleming et al., 1996). The recovery of both sugars was determined per litre serum (mg L^−1^). Calculation of the L/R ratio was corrected relative (%) to the concentration of sugar consumed. The limit of detection was 0.1 mg L^−1^ and the laboratory reference coefficient of variation was 1.8%–8.5% for both probes (Fleming et al., 1996).

### 2.10 Enzyme linked immunosorbent assays

I‐FABP ([1:2 serum dilution]; ELH‐FABP2, Raybiotech®), CLDN‐3 ([undiluted plasma]; EH1342, Wuhan Fine Biotech) and LBP ([1:250 plasma dilution]; RK01764, ABcloncal) were measured in duplicate immediately pre‐ and post‐EHST using a solid‐phase sandwich ELISA. Optical density was measured at 450 nm using a microplate reader and sample concentrations were determined from a logarithmic standard curve. The intra‐assay coefficients of variation were 5.0% (I‐FABP), 1.5% (CLDN‐3), and 2.6% (LBP).

### 2.11 Quantitative real‐time polymerase chain reaction

BactDNA was measured in duplicate plasma samples collected immediately pre‐ and post‐EHST using a quantitative real‐time polymerase chain reaction assay (qPCR) on a LightCycler 96 instrument (LightCycler 96, Roch). Cell‐free DNA was isolated from plasma using a Quick‐DNA Mini Prep Plus kit (D4068, Zymo Research) following manufacturer's instructions. Total 16S bacterial DNA was quantified according to March et al. (2019) using a universal library probe (ULP, Roche, Basel, Switzerland), with standards (E2006‐2, Zymo Research) and primers (Eurogentec, Liège, Belgium) specific to a 16S region (limit of detection 0.1 pg µl^−1^). *Bacteroides* species DNA (*Bact.* DNA) were quantified using a double‐dye probe/primer kit (Path‐Bacteroides‐spp, Genesig, Primerdesign Ltd). Negative controls (PCR grade water) for the entire extraction process were below the limit of detection for both measures. Ratio data are presented as *Bacteroides*/total bacterial DNA (*Bact./*16S). The intra‐assay coefficients of variation were 6.3% (total 16S) and 17.5% (*Bacteroides*).

### 2.12 Statistics

All statistical analyses were performed using Prism Graphpad software (Prism V.8, La Jolla, California, USA). Comparisons were made after determining normal distribution using a Shapiro–Wilk test (*p* ≥ .05). A two‐way analysis of variance (ANOVA) with repeated measures (time x trial) was used to identify differences between the two trials for whole‐body physiological, GI barrier integrity, and MT data. If Mauchly's test for sphericity was violated, Greenhouse Geiser corrections were applied for epsilon <0.75, while the Huynh–Feldt correction was used for less severe asphericity. When there was only a single comparison, a paired *t* test or nonparametric Wilcoxon signed‐ranks test was used to determine between‐trial differences. Statistical significance was accepted at the alpha level of *p* ≤ .05. Data are presented as mean ± standard deviation (*SD*).

A composite a priori battery of statistical tests was conducted to determine intertrial reliability (Atkinson & Nevill, 1998). The DSAT was compared at each 90‐ and 150‐min following sugar‐probe ingestion, while each GI biomarker was compared at rest, post‐EHST and the delta (Δ). Systematic bias was assessed using a paired *t* test or nonparametric Wilcoxon signed‐ranks test. Meaningful differences were evaluated using Cohen's *d* (Lakens, 2013). Effect sizes were categorized as trivial (≤0.19), small (0.20–0.49), medium (0.50–0.79), and large (≥0.8). Relative reliability was assessed using a Pearson's product‐moment correlation coefficient or nonparametric Spearman's rank correlation coefficient. Correlations were classified as small (≤0.69), moderate (0.70–0.89) and high (≥0.90) (Vincent & Weir, 1995). Absolute reliability was assessed using each of the coefficient of variation ([*SD*/mean]*100), typical error of the measurement (TEM; *SD* of difference between scores/√2) and Bland–Altman (B‐A) plots with mean difference (bias), and 95% Limits of Agreement (LoA; Bland & Altman, 1986). CVs were classified as very good (≤10%) and acceptable (≤20%). Relationships between biomarkers were compared using a Pearson's product–moment correlation coefficient or nonparametric Spearman's rank correlation coefficient. Heteroskedasticity was examined from the nonparametric correlational coefficient between absolute differences and individual means presented on B‐A plots. Outliers were defined as ±2.4 *SD* units (normally distributed) or ±4.0 *SD* units (nonnormally distributed) outside of the mean and were removed from subsequent analysis (Aguinis, Gottfredson, & Joo, 2013).

### 2.13 Power analysis

Given the novelty of the dependent variables being evaluated and statistical approach to undertake a battery of reliability statistical tests, it was determined infeasible to perform an a priori sample size calculation. Instead, general guidance on appropriate sample sizes (*n* = 12) for pilot studies were followed, while accounting for a ~20% anticipated participant drop‐out rate (Julious, 2005).

## 3 RESULTS

### 3.1 Thermoregulatory, cardiovascular, and perceptual strain

**Figure 2:**
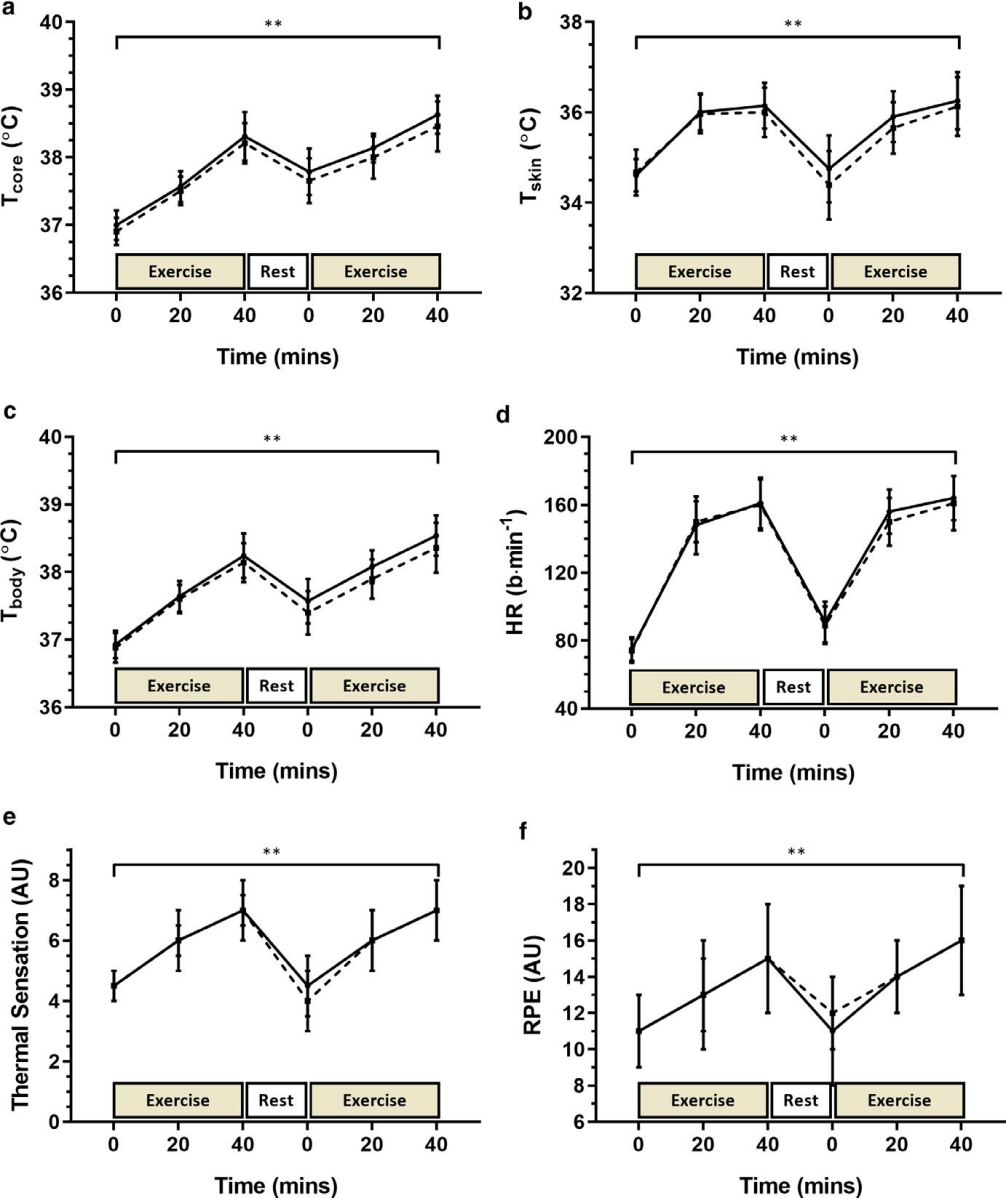
Whole‐body physiological responses to repeated EHSTs: (a) = core temperature; (b) = mean skin temperature (*n* = 13); (c) = mean body temperature (*n* = 13); (d) = heart rate; (e) = thermal sensation; and (f) = rate of perceived exertion. Solid line = trial 1, broken line = trial 2. Significant overall effect of time (*p ≤ .05; **p ≤ .01)

T_core_ (Figure 2a; time x trial *p* = .63), T_skin_ (Figure 2b; time x trial *p* = .13) and T_body_ (Figure 2c; time × trial *p* = .43) all increased over time to a similar extent between trial one and two. The reliability of peak, mean, and Δ in T_core_, T_skin_ and T_body_ were all good (Table 2). Pretrial plasma osmolality (trial one: 293 ± 7 mOsmol kg^−1^, trial two: 294 ± 7 mOsmol kg^−1^; *p* = .67), Δ plasma volume (trial 1: −0.61 ± 5.15%, trial 2: −0.02 ± 3.69%; *p* = .67), mean sweat rate (trial 1: 1.53 ± 0.38, trial 2:1.56 ± 0.45 L h^−1^; *p* = .61) and percentage body mass loss (trial 1: 1.15 ± 0.48; trial 2: 1.21 ± 0.52%; *p* = .31) were all similar between trial one and two. HR (Figure 2d; time x trial *p* = .11), RPE (Figure 2e; time x trial *p* = .38) and TS (Figure 2f; time × trial *p* = .56) all increased over time to a similar extent between trial one and two. The reliability of peak, mean, and Δ HR, RPE, and TS were all good (Table 2).

**Table 2:** Relative and absolute reliability of whole‐body physiological responses

	Trial 1 (*SD*)	Trial 2 (*SD*)	*p*	*d*	*r*	CV	TEM	Bias (LoA)
T_core_ (°C) Peak	38.63 ± 0.28	38.46 ± 0.37	.02	0.49	0.78**	0.5	0.14	0.17 ± 0.46
T_core_ (°C) Mean	37.87 ± 0.19	37.78 ± 0.23	.09	0.43	0.68**	0.3	0.13	0.09 ± 0.43
T_core_ (°C) Δ	1.62 ± 0.29	1.55 ± 0.44	.59	0.19	0.48	—	0.26	0.07 ± 0.77
T_skin_ (°C) Peak	36.27 ± 0.63	36.13 ± 0.65	.23	0.22	0.83**	0.6	0.27	0.13 ± 0.74
T_skin_ (°C) Mean	35.75 ± 0.44	35.64 ± 0.49	.13	0.24	0.83**	0.4	0.17	0.11 ± 0.46
T_skin_ (°C) Δ	1.65 ± 0.63	1.46 ± 0.65	.18	0.30	0.71**	—	0.35	0.20 ± 0.97
T_body_ (°C) Peak	38.54 ± 0.30	38.36 ± 0.37	.01	0.53	0.79**	0.4	0.16	0.18 ± 0.44
T_body_ (°C) Mean	37.83 ± 0.20	37.74 ± 0.23	.08	0.42	0.70**	0.3	0.12	0.09 ± 0.33
T_body_ (°C) Δ	1.62 ± 0.30	1.49 ± 0.40	.18	0.37	0.60*	—	0.23	0.13 ± 0.63
HR (b·min^−1^) Peak	164 ± 13	162 ± 14	.19	0.15	0.93**	2.0	4	2 ± 11
HR (b·min^−1^) Mean	150 ± 14	148 ± 13	.29	0.14	0.84**	2.8	5	2 ± 15
HR (b·min^−1^) Δ	90 ± 11	90 ± 13	.99	0.00	0.88**	—	4	0 ± 12
RPE (AU) Peak	16 ± 3	16 ± 3	.99	0.00	0.92**	4.1	1	0 ± 3
RPE (AU) Mean	13 ± 2	13 ± 2	.88	0.00	0.80**	5.2	1	0 ± 2
RPE (AU) Δ	6 ± 5	5 ± 3	.95	0.24	0.72**	—	2	0 ± 4
TS (AU) Peak	7.0 ± 0.5	7.0 ± 0.5	.99	0.00	0.84**	4.8	0.5	0.0 ± 1.0
TS (AU) Mean	6.0 ± 0.5	5.5 ± 0.5	.02	0.00	0.88**	3.2	0.0	0.5 ± 0.5
TS (AU) Δ	2.5 ± 1.0	2.5 ± 0.5	.12	0.00	0.61*	—	0.5	−0.5 ± 1.0

* Significant correlation (*p* ≤ .05);

** Significant correlation (*p* ≤ .01).

### 3.2 Dual‐Sugar Absorption Test

**Figure 3:**
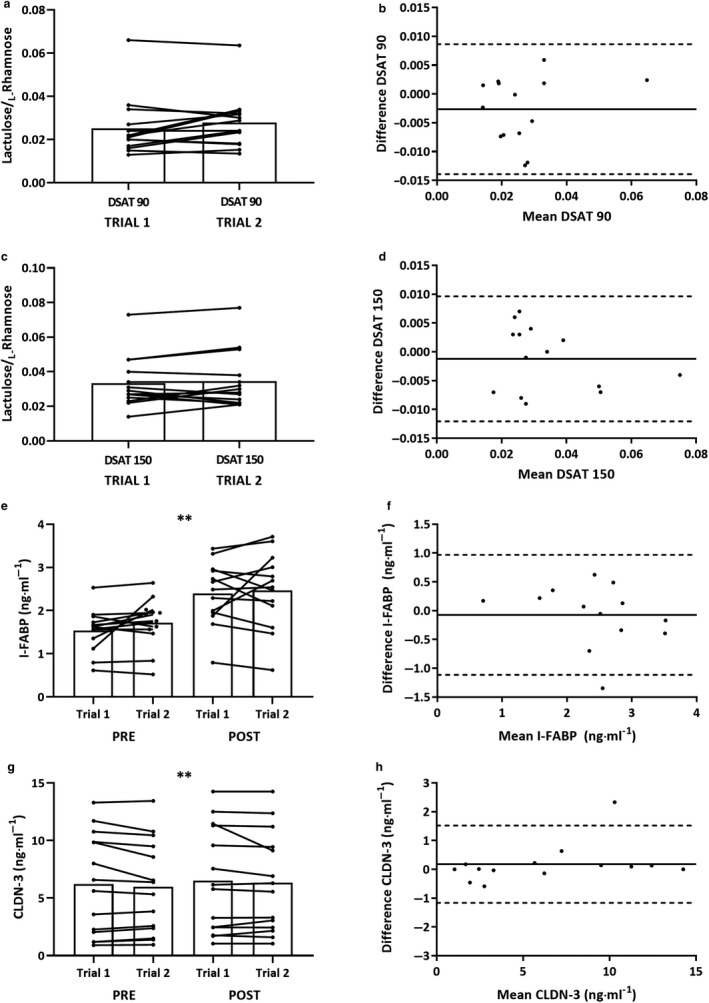
GI barrier integrity responses to EHST trial 1 and trial 2: (a) = L/R ratio at 90 min; (c) = L/R ratio at 150 min; (e) I‐FABP (*n* = 13); and (g) = CLDN‐3. Significant overall effect of time (*p ≤ .05; **p ≤ .01). Bland–Altman mean bias and 95% LoA between post‐EHST trial 1 and trial 2: (b) = L/R ratio at 90‐ and (d) 150‐ min; (F) I‐FABP (*n* = 13); and (h) = CLDN‐3

Lactulose and _L_‐rhamnose were both undetectable in all participants’ basal sample prior to probe ingestion. Inter‐trial DSAT responses displayed no systematic bias between trials at both 90‐ (Figure 3a) and 150‐min (Figure 3c). There was moderate relative reliability and acceptable absolute reliability at both the 90‐ and 150‐min time‐point. B–A plots displayed bias for both the 90‐ (Figure 3b) and 150‐min (Figure 3d) time‐point. Individual Lactulose and _L_‐rhamnose concentrations had worse reliability than the combined L/R ratio (Table 3). Heteroskedasticity was not present for any analyses.

**Table 3:** Relative and absolute reliability of all GI barrier integrity biomarkers

	Trial 1 (*SD*)	Trial 2 (*SD*)	*p*	*d*	*r*	CV	TEM	Bias (LoA)
Lactulose (mg·L^−1^) 90	1.06 ± 0.38	0.90 ± 0.43	.33	0.38	0.60*	21.3	0.263	0.151 ± 0.730
_L_‐Rhamnose (mg·L^−1^) 90	15.89 ± 3.91	15.85 ± 3.13	.29	0.29	0.53	12.9	2.601	1.036 ± 6.930
DSAT (L/R) 90	0.028 ± 0.012	0.025 ± 0.014	.17	0.23	0.77**	11.5	0.004	−0.003 ± 0.011
Lactulose (mg·L^−1^) 150	0.97 ± 0.48	0.95 ± 0.52	.53	0.05	0.71**	13.0	0.132	0.023 ± 0.364
_L_‐Rhamnose (mg·L^−1^) 150	12.01 ± 2.95	11.24 ± 2.96	.09	0.27	0.86**	7.6	1.149	0.772 ± 3.060
DSAT (L/R) 150	0.033 ± 0.015	0.034 ± 0.016	.37	0.06	0.69**	12.2	0.004	0.001 ± 0.011
I‐FABP (ng·ml^−1^) Rest	1.560 ± 0.506	1.691 ± 0.555	.11	0.25	0.75**	11.1	0.304	−0.180 ± 0.746
I‐FABP (ng·ml^−1^) Post	2.394 ± 0.731	2.467 ± 0.875	.63	0.09	0.80**	12.1	0.376	−0.073 ± 1.040
I‐FABP (ng·ml^−1^) Δ	0.834 ± 0.445	0.776 ± 0.489	.65	0.12	0.65**	—	0.278	0.058 ± 0.772
CLDN−3 (ng·ml^−1^) Rest	6.205 ± 4.382	5.971 ± 4.062	.17	0.06	0.99**	6.8	0.423	0.233 ± 1.172
CLDN−3 (ng·ml^−1^) Post	6.592 ± 4.770	6.323 ± 4.270	.34	0.06	0.99**	4.9	0.485	0.181 ± 1.341
CLDN−3 (ng·ml^−1^) Δ	0.317 ± 0.586	0.371 ± 0.508	.68	0.10	0.62*	—	0.342	−0.055 ± 0.948

* Significant correlation (*p* ≤ .05);

** Significant correlation (*p* ≤ .01).

### 3.3 Intestinal fatty acid‐binding protein

I‐FABP displayed no trial order systematic bias at either rest, post‐ or the Δ time‐point (Figure 3e). Following EHSTs, I‐FABP was elevated above rest (trial 1: Δ = 0.834 ± 0.445 ng·ml^‐1^ [56 ± 31%]; trial 2: Δ = 0.776 ± 0.489 ng ml^−1^ [46 ± 26%]; *p* ≤ .01; Figure 3e). At all‐time‐points, I‐FABP displayed moderate relative and acceptable absolute reliability (Table 3). B–A plots are presented to illustrate bias for post‐EHST concentrations (Figure 3f). Heteroskedasticity was not present for any analyses. One participant was excluded as an outlier. Two participants’ I‐FABP responses displayed unexplainably poor reliability both at rest and post‐EHSTs. These data were retained given where verbal adherence to pretrial controls was verbally confirmed. However, removal of these data would have notably improved the reliability of I‐FABP both at rest (*r* = 0.97; CV = 6.1%; TEM = 0.200 ng ml^−1^; B‐A ± LoA = −0.046 ± 0.308 ng ml^−1^), and post‐EHST (*r* = 0.97; CV = 7.2%; TEM = 0.221 ng ml^−1^; B‐A ± LoA = 0.078 ± 0.467 ng ml^−1^).

### 3.4 Claudin‐3

CLDN‐3 displayed no trial order systematic bias at either rest, post‐ or the Δ time‐point (Figure 3g). Following EHSTs CLDN‐3 was elevated above rest (trial 1: Δ = 0.317 ± 0.586 ng ml^−1^ [11 ± 17%]; trial 2: Δ = 0.371 ± 0.508 ng ml^−1^ [9 ± 13%]; *p* ≤ .01; Figure 3g). At all time‐points, CLDN‐3 displayed high relative and very good absolute reliability (Table 3). B‐A plots are presented to illustrate bias for post‐EHST concentrations (Figure 3h). Heteroskedasticity was not present for any analyses.

### 3.5 Lipopolysaccharide‐binding protein

**Figure 4:**
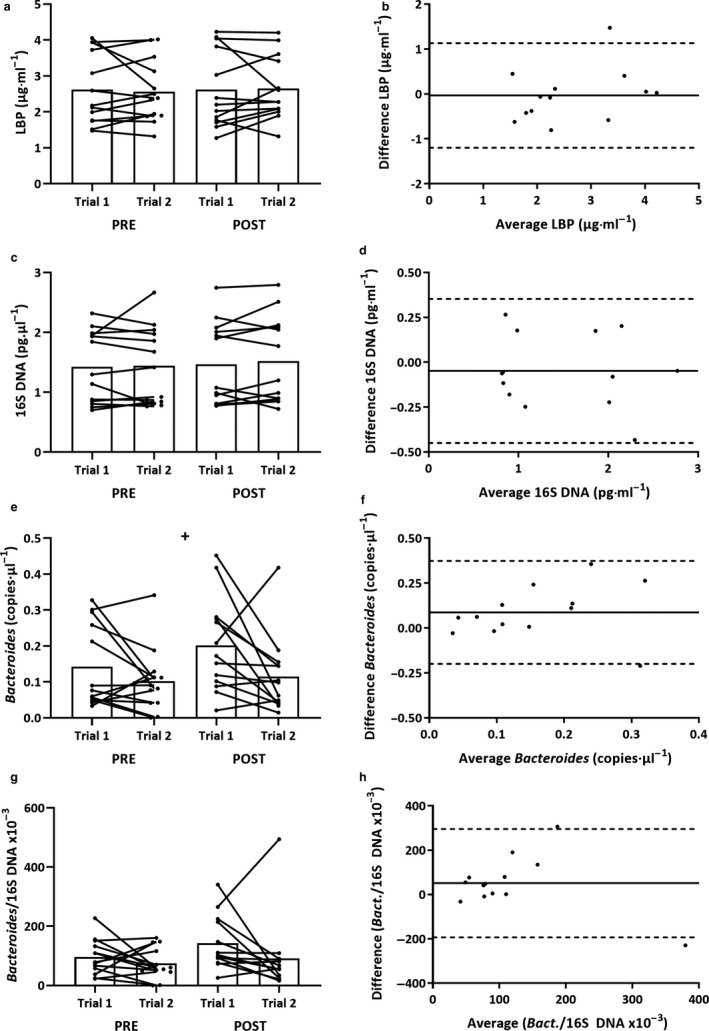
GI MT responses to EHST trial 1 and trial 2: (a) = LBP; (c) total 16S bacterial DNA (*n* = 13); (e) = *Bacteroides* DNA; and (g) = *Bacteroides*/total 16s bacterial DNA (*n* = 13). Significant overall effect of trial (+*p* ≤ .05). Bland‐Altman mean bias and 95% LoA between post‐EHST trial 1 and trial 2: (b) = LBP; (d) = total 16S bacterial DNA (*n* = 13); (f) *Bacteroides* DNA (*n* = 13); and (h) = *Bacteroides*/total 16s bacterial DNA (*n* = 13)

LBP displayed no trial order systematic bias at either rest, post‐EHSTs or the Δ time‐point (Figure 4a). There was no influence of the EHST on LBP concentration (*p* = .41). At all time‐points, LBP displayed moderate relative and very good absolute reliability (Table 4). B‐A plots are presented to illustrate bias for post EHST concentrations (Figure 4b). Heteroskedasticity was not present for any analyses.

**Table 4:** Relative and absolute reliability of all GI barrier integrity biomarkers

	Trial 1 (*SD*)	Trial 2 (*SD*)	*p*	*d*	*r*	CV	TEM	Bias (LoA)
LBP (µg·ml^−1^) Rest	2.625 ± 0.993	2.564 ± 0.871	.79	0.07	0.85**	10.0	0.378	0.061 ± 1.048
LBP (µg·ml^−1^) Post	2.617 ± 1.080	2.650 ± 0.885	.59	0.03	0.85**	9.2	0.420	−0.033 ± 1.166
LBP (µg·ml^−1^) Δ	−0.008 ± 0.250	0.086 ± 0.232	.38	0.39	−0.16	—	0.260	−0.094 ± 0.721
16S DNA (pg·µl^−1^) Rest	1.43 ± 0.60	1.44 ± 0.65	.79	0.02	0.87**	8.1	0.19	−0.02 ± 0.49
16S DNA (pg·µl^−1^) Post	1.47 ± 0.70	1.52 ± 0.73	.45	0.07	0.82**	9.5	0.15	−0.05 ± 0.40
16S DNA (pg·µl^−1^) Δ	0.04 ± 0.28	0.08 ± 0.40	.72	0.10	0.56*	—	0.24	−0.03 ± 0.65
*Bact.* DNA (copies·µl^−1^) Rest	0.142 ± 0.116	0.102 ± 0.089	.19	0.39	0.14	55.0	0.067	0.040 ± 0.186
*Bact.* DNA (copies·µl^−1^) Post	0.202 ± 0.132	0.115 ± 0.106	.04*	0.73	0.14	56.3	0.104	0.087 ± 0.287
*Bact.* DNA (copies·µl^−1^) Δ	0.059 ± 0.149	0.013 ± 0.140	.22	0.32	0.61*	—	0.091	0.047 ± 0.250
*Bact.*/16S DNA (x10^−3^) Rest	96.08 ± 59.31	74.10 ± 52.82	.31	0.39	0.13	60.2	52.49	−0.019 ± 0.348
*Bact.*/16S DNA (x10^−3^) Post	143.41 ± 90.73	91.83 ± 124.76	.07	0.47	0.20	54.7	88.18	0.032 ± 0.347
*Bact.*/16S DNA (x10^−3^) Δ	47.32 ± 95.74	17.74 ± 138.78	.35	0.25	0.69*	—	76.88	0.052 ± 0.378

* Significant correlation (*p* ≤ .05);

** Significant correlation (*p* ≤ .01).

### 3.6 Bacterial DNA

Total 16s (Figure 4c) and *Bact.*/16S (Figure 4g) displayed no systematic bias at either rest, post EHSTs or the Δ. *Bacteroides* concentrations (Figure 4e) were systematically lower in trial 2 versus trial 1 (*p* = .04). At rest, total 16s displayed moderate relative and very good absolute reliability, whereas *Bacteroides* displayed poor relative and absolute reliability. The combined *Bact.*/16S ratio subsequently showed poor relative and absolute reliability at rest (Table 4). There was no influence of the EHST on either total 16s (*p* = .39), *Bacteroides* (*p* = .33) or *Bact.*/16S (*p* = .18) responses. B–A plots are presented to illustrate bias for post‐EHST concentrations (Figure 4d,f,h). Heteroskedasticity was not present for any analyses. One participant was excluded as an outlier.

### 3.7 Association between biomarkers

Validation of the DSAT at the 90 and 150‐ min time‐points across both trial one and trial two (*n* = 28), found responses to be systematically greater at 150‐ (0.034 ± 0.015) compared with 90 min (0.027 ± 0.013; *p* = .05, ES 0.50). There was poor relative (*r* = 0.08) and absolute (CV = 31.8%, TEM = 0.014) reliability between the two DSAT sample points, suggestive of interindividual variability in sugar probe kinetics. Few statistically significant correlations were reported when comparing GI barrier integrity and MT biomarkers. Small positive correlations were reported between absolute post‐EHST concentrations for I‐FABP and CLDN‐3 (*r* = 0.41, *p* = .04), LBP and total 16s DNA (*r* = 0.48, *p* = .02), LBP and *Bacteroides* (*r* = 0.38; *p* = .05), *Bacteroides*, and total 16s DNA (*r* = 0.40, *p* = .04). When displayed as pre‐to‐post‐DELTA, small positive correlations were reported between LBP and DSAT at 150 min (*r* = 0.54; *p* < .01).

## 4 DISCUSSION

The aim of this study was to determine the short‐term (1–2 weeks) temporal reliability of several empirical biomarkers of GI barrier integrity (DSAT, I‐FABP, CLDN‐3) and MT (LBP, total 16s bacterial DNA, *Bacteroides* DNA) following exertional‐heat stress. The main findings of this study were that the serum DSAT, I‐FABP, CLDN‐3, LBP and total 16s bacterial DNA all displayed moderate‐to‐strong relative and acceptable absolute reliability between repeat EHSTs. In comparison, absolute *Bacteroides* DNA and *Bact./*total 16s DNA ratio displayed weak relative and unacceptable absolute reliability between repeat EHSTs.

The serum DSAT is a valid alternative of the traditional urine DSAT (Fleming et al., 1996; van Wijck et al., 2011), whilst offering improved sensitivity to detect transient losses in GI barrier integrity following exercise (JanssenDuijghuijsen et al., 2016; Pugh et al., 2017). Despite this, the temporal reliability of the serum DSAT has never been previously assessed. Potential sources of variability with the serum DSAT might relate to both the transient time course of sugar probes in the blood and low absolute lactulose concentration that challenge the detection limit of common analytical techniques (Fleming et al., 1996; van Wijck et al., 2013). In this study, we show for the first time that the serum DSAT can be utilized with acceptable reliability, which is comparative to that previously reported with the urine DSAT when repeated over both a three‐day (van Elburg et al., 1995) and two‐week period (Marchbank et al., 2010). The optimal time‐point for blood collection with the serum DSAT is an unresolved issue that concerns the methodological implementation of this measure. Herein, blood was collected at both 90‐min postprobe ingestion as this provides the most valid estimate of the urine DSAT in basal conditions (Fleming et al., 1996), and at 150‐min post as this is where peak responses arose following similar exercise stress (van Wijck et al., 2011). Remarkedly, the temporal reliability of both time‐points assessed was almost identical, though given large interindividual variation in probe kinetics, the magnitude of responses at the two time‐points had poor validity. Together, these findings advocate the use of the serum DSAT at either 90 or 150 min following probe ingestion (where logistically most convenient) as a reliable alternative to the urine DSAT.

I‐FABP is the principal biomarker of GI epithelial injury (Wells et al., 2017). Despite growing popularity, the temporal reliability of circulating I‐FABP has never been previously assessed. In the present study, resting I‐FABP concentrations were consistently at the upper end of the general healthy reference range for studies utilizing a human ELISA kit (0.1–2.0 ng ml^−1^; Treskes, Persoon, & Zanten, 2017). These concentrations must be considered when evaluating the absolute reliability thresholds reported herein. The rationale for large between‐study discrepancies in absolute I‐FABP concentrations are poorly understood, though are more likely attributable to analytical discrepancies (e.g. ELISA antibody, ELISA wash procedure, sample storage), than participant demographic (Treskes et al., 2017). The reliability of I‐FABP at rest displayed moderate relative and acceptable absolute reliability. Following both EHSTs, I‐FABP increased by approximately 50% or 0.800 ng ml^−1^. This response is comparable to numerous similar duration/intensity exercise protocols, such as: 45‐to‐60 min of ~70% watt_max_ normothermic cycling (van Wijck et al., 2011, [61%, Δ 0.306 ng ml^−1^] 2012, [61%; Δ 0.179 ng ml^−1^] and 20–30 min of ~80% VO_2max_ running (Barberio et al., 2015 [46%, Δ 0.297 ng ml^−1^]; March et al., 2017 [72%; Δ 0.350 ng ml^−1^]). In comparison, far greater elevations in I‐FABP have been shown following 90–120 min of moderate‐intensity running performed in the heat (30°C; Morrison, Cheung, & Cotter, 2014 [663%; Δ 0.203–0.806 ng ml^−1^]; Snipe et al., 2017 [288%, Δ 0.897 ng ml^−1^]; 2018 [432%, Δ 1.230 ng ml^−1^]. Given the high sensitivity of I‐FABP to even minor GI injury, it is vital that known extraneous variables (e.g., prandial/hydration status, prior exercise) are tightly controlled prior to investigation. Whilst participants in the present study provided written conformity to all pretrial controls, two participants’ resting I‐FABP concentrations appeared suspect to prior GI injury in one trial, which interestingly was unable to be detected by any other analyte.

CLDN‐3 is the principle biomarker of GI TJ integrity (Wells et al., 2017). Despite introduction as a TJ biomarker almost a decade ago, the biological relevance of elevated circulating CLDN‐3 is still poorly understood. This includes the assessment of temporal reliability, which is currently unknown. In the present study, resting CLDN‐3 concentrations were consistent with previous evidence (0.5–15 ng ml^−1^) in healthy populations (Typpo et al., 2015; Yeh, Law, & Lim, 2013). At rest, large interindividual variation in CLDN‐3 concentration was evident, meaning that relative reliability was almost uniform. Following both EHSTs, plasma CLDN‐3 consistently increased by approximately 8%–10%. This finding compares well to the only previous exercise study, where concentrations increased directly following a 1 hr moderate‐intensity (70% VO_2max_) run in both temperate (22°C; 6.7 > 7.6 ng ml^−1^) and hot (33°C; 6.6 > 8.2 ng ml^−1^) ambient environments (Yeh et al., 2013). The clinical relevance of this small, transient increase in CLDN‐3 following exercise is poorly understood, though it is modest in comparison with the magnitude of increase (4–20‐fold) shown acutely following major nonabdominal surgery (Habes et al., 2017; Typpo et al., 2015). Promisingly, of all the GI barrier integrity biomarkers compared, CLDN‐3 displayed the strongest relative and absolute reliability.

LBP is a type‐1 acute phase protein that responds to a wide variety of microbial‐associated molecular patterns and is widely considered a stable indirect biomarker of bacterial endotoxin exposure (Dullah & Ongkudon, 2017). In the present study, resting LBP concentrations displayed showed moderate relative and good absolute reliability. These results are in support of one previous study, which found short‐term (≤7 day) basal LBP responses to display moderate relative reliability (intraclass correlation coefficient = 0.61; Citronberg et al., 2016). In comparison, direct assessment of endotoxin appears to have weak basal temporal reliability, with an intraindividual CV of 22% reported over a similar 7‐day period in basal conditions (Guy, Edwards, Miller, Deakin, & Pyne, 2017). Following the EHST, LBP was unchanged in both trials, with concentrations offering comparable levels of reliability compared to rest. While the evidence is sparse regarding LBP responses to exercise, previous evidence has shown a minor elevation in LBP of 10%–15% immediately following a fatiguing treadmill walk (4.5 km hr^−1^) in the heat (40°C; 106 min; Selkirk, McLellan, Wright, & Rhind, 2008), and 1 hr of moderate intensity (70% VO_2max_) treadmill running (Jonvik et al., 2019). A potential explanation for these discrepant findings likely relate to greater thermoregulatory/cardiovascular strain in previous studies.

BactDNA is an emerging GI MT biomarker, given the recent characterization of the blood microbiome and improvements in 16S PCR sensitivity (Paisse et al., 2016). In the present study, resting total 16S DNA concentrations displayed moderate relative and good absolute reliability. This finding is promising, given previous concerns that plasma bactDNA concentrations are susceptible to contamination during sample analysis (Glassing, Dowd, Galandiuk, Davis, & Chiodini, 2016). Quantification of total plasma 16S bacterial DNA in exercise settings has never been previously examined, though consistent with other MT biomarkers, the present results show total 16S bactDNA to be stable following moderate intensity exertional heat stress. One criticism of total 16S bactDNA assessment, particularly in exercise settings, is a lack of GI specificity, with total concentrations influenced by factors, including DNase concentration (Velders et al., 2014) and 16S DNA contamination from other body blood compartments (Paisse et al., 2016). To account for this error, one hypothetically improved method involves targeting a highly abundant GI genus such as *Bacteroides* (~30% of GI microbiota) and correcting for total 16S concentration (March et al., 2019). This method is particularly favorable given that the phyla *Firmicutes* and *Bacteroidetes* comprise >90% of the GI microbiome (*Bacteroides*; Huttenhower et al., 2012) and <5% of the plasma microbiome (Paisse et al., 2016). Utilizing this hypothesis, March et al. (2019) reported that the plasma *Bacteroides*/16S DNA ratio tended to increase (~25%; *p* = .07) following a 1‐hr moderate intensity (70% VO_2max_) run in the heat (30°C), though large interindividual variability in responses were evident. In this study, the *Bacteroides*/16S DNA ratio was unchanged following the EHST and appeared to be systematically lower following exercise in trial two (but not the Δ). This systematic bias was unexpected given the uniformity of all other analytes assessed and the poor analytical reliability of this biomarker (e.g., mean duplicate CV = 17.5%). It is presently unclear whether the poor reliability of this measure has obscured a true effect of the EHST and/or the meaningfulness of this variability during more severe MT.

Evidence directly comparing correlations between GI barrier integrity and/or MT biomarkers in exercise settings has been limited to date. Given general logistical constraints of the urine/serum DSAT, the majority of relevant evidence has attempted to validate (correlate) this method against more practical GI barrier integrity biomarkers. These studies generally report significant, though weak correlations (*r* = 0.4–0.6) between basal corrected (Δ) DSAT (urine 5 hr) and I‐FABP responses directly following moderate GI barrier integrity loss (March et al., 2017; van Wijck et al., 2011, 2012). In this study, the DSAT did not correlate with any other GI integrity biomarker. A potential explanation for this null finding might result from the lack of basal DSAT correction or the low overall severity of GI barrier integrity loss. In comparison, a small positive correlation was reported between I‐FABP and CLDN‐3. This finding is supportive of previous evidence showing urinary I‐FABP and CLDN‐3 to weakly correlate (*r* = 0.38) in patients with major nonabdominal surgery (Habes et al., 2017). The expression of CLDN‐3 across multiple tissues might partially explain why this correlation was not stronger (Thuijls, Derikx, Haan, et al., 2010a). In general, no GI barrier integrity and MT biomarkers, except DSAT 150 and Δ LBP, were found to correlate. Previous exercise gastroenterology research has shown various combinations of these biomarkers to correlate weakly (*r* = 0.1–0.6; Yeh et al., 2013; Sessions et al., 2016; March et al., 2019) or not at all (Karhu et al., 2017; Snipe, Khoo, Kitic, Gibson, & Costa, 2018). Several physiological (e.g. hepatic/immune microbial clearance, transcellular microbial translocation, GI microbial density), and analytical (e.g., exogenous sample contamination, inconsistent biomarker kinetics, DSAT/I‐FABP limited to small GI integrity) factors all likely weaken these associations (Wells et al., 2017).

## 5 LIMITATIONS

Despite implementation of a tightly controlled methodological design, which accounted for the majority of extraneous variables, the presented results are not without some limitations. First, the EHST was only able to evoke moderate GI barrier integrity loss and did not influence MT, potentially limiting the application of these findings in severe situations of GI barrier integrity loss. A previous systematic review has suggested an exercise‐induced hyperthermia threshold of 38.6°C T_core_ for GI barrier integrity loss (DSAT, I‐FABP and endotoxin) to be commonplace (>50% incidence) and of 39.0°C for GI barrier integrity loss to be universal (100% incidence; Pires et al., 2017). Consistently, previous research supports the notion that MT biomarkers (endotoxin) are less responsive to subtle alterations in GI barrier integrity that were otherwise detected by the DSAT or I‐FABP following exercise (March et al., 2019; Snipe et al., 2017, 2018). Positively, no GI barrier integrity or MT biomarker displayed statistical heteroskedasticity in this EHST model, suggestive that absolute reliability was not dependent upon the magnitude of biomarker response. Next, biomarker analysis was limited to a single time‐point after the EHST (at termination), though this can be justified in that peak responses are consistently shown to occur at this instance in comparable exertional heat stress interventions (e.g., *I‐FABP*, Snipe et al., 2017; *CLDN‐3*, Yeh et al., 2013; *LBP*, Moncada‐Jimenez et al., 2010; *Bact./16S*, March et al., 2019). Third, there was statistically significant systematic bias for peak T_core_ and T_body_ responses, which were lower (0.17°C and 0.18°C) following implementation of trial two. This result was not anticipated, given numerous previous studies showing a one week washout period to be sufficient in preventing carry‐over (heat acclimation) effects following exertional heat stress exposure of comparable severity (Barrett and Maughan, 1993; Willmott et al., 2015). The meaningfulness of this systematic bias does not appear to statistically influence any GI barrier integrity of MT biomarker. Finally, neither a basal DSAT or urinary DSAT were performed, consequently preventing direct determination of the EHST on DSAT results or allowing comparisons between DSAT responses across biofluids. This decision was made to minimize participant time burden.

## 6 CONCLUSION

This is the first study to comprehensively assess the reliability of GI barrier integrity and/or microbial translocation biomarkers both at rest and following exertional (‐heat) stress. Quantifying biomarker reliability is a vital step required to inform marker selection for application in laboratory and field settings. Each of the GI barrier integrity biomarkers assessed displayed moderate‐to‐good relative and acceptable absolute reliability both at rest and post‐ EHST. Serum DSAT responses had comparable reliability at two separate time‐points following sugar‐probe ingestion (90‐ and 150‐min), though response kinetics displayed inconsistent time courses. I‐FABP and CLDN‐3 both increased following the EHST and their responses were found to weakly correlate. None of the selected microbial translocation biomakers were elevated following the EHST, suggestive that a greater severity of GI barrier integrity loss is required for MT. LBP and total 16S DNA both demonstrated moderate‐to‐good relative and acceptable absolute reliability at both time‐points. There was a weak correlation between LBP and total 16S post‐EHST responses. Despite offering superior methodological rationale, *Bacteroides* DNA or *Bacteroides*/total 16S DNA had unacceptable reliability. The findings of the present study have direct relevance for evaluating the efficacy of interventions to attenuate the rise in GI barrier integrity/MT when exercising in the heat. Such interventions might include exercise training, heat acclimatization and nutritional supplementation. The findings of this study might also have value to the pharmaceutical industry, to quantify the efficacy of drugs to maintain GI barrier integrity, or to evaluate improvements in drugs that traditionally resulted in GI barrier integrity loss.

## CONFLICT OF INTERESTS

No competing interests.

## AUTHOR CONTRIBUTION

HO, JF, RC, SD, CW, and JL concepted and designed the research; HO, AM, CW performed the experiments; HO, GD, SF and RE acquired data; HO, JF, RC, GD, SD, and JL interpreted the results; HO wrote the manuscript; HO, JF, RC, GD, SF, RE, AM, CW, AM, and JL edited, revised and agree to accountability of the accuracy and integrity of the manuscript. Data were collected at School of Sport, Health and Wellbeing, Plymouth MARJON University. All persons designated as authors qualify for authorship, and all those who quality for authorship are listed.
